# *miR-27a* and *miR-449b* polymorphisms associated with a risk of idiopathic recurrent pregnancy loss

**DOI:** 10.1371/journal.pone.0177160

**Published:** 2017-05-10

**Authors:** HyungChul Rah, Ki Wha Chung, Ki Han Ko, Eun Sun Kim, Jung Oh Kim, Jung Hyun Sakong, Ji Hyang Kim, Woo Sik Lee, Nam Keun Kim

**Affiliations:** 1 Department of Business Data Convergence, Chungbuk National University, Cheongju, South Korea; 2 Department of Biological Science, College of Natural Science, Kongju National University, Gongju, South Korea; 3 Department of Biomedical Science, College of Life Science, CHA University, Seongnam, South Korea; 4 Department of Obstetrics and Gynecology and Fertility Center of CHA Bundang Medical Center, CHA University, Seongnam, South Korea; 5 Fertility Center of CHA Gangnam Medical Center, CHA University, Seoul, South Korea; 6 Institute for Clinical Research, CHA Bundang Medical Center, CHA University, Seongnam, South Korea; Institut de Pharmacologie Moleculaire et Cellulaire, FRANCE

## Abstract

**Objective:**

MicroRNAs (miRNAs) regulate gene expression during the peri-implantation period. The purpose of this study was to investigate whether genetic polymorphisms in the four miRNAs associated with fetal or placental development play roles in the development of idiopathic recurrent pregnancy loss (RPL) in Korean females.

**Study design:**

A case-control study involving 225 controls and 387 women with at least two consecutively recurrent pregnancy losses between 1999 and 2012 was performed. The genotypes of the four miRNA polymorphisms, including *miR-27a* rs895819, *miR-423* rs6505162, *miR-449b* rs10061133, and *miR-605* rs2043556, were analyzed by the polymerase chain reaction-restriction fragment length polymorphism assay. Odds ratios and 95% confidence intervals were estimated using multivariate analyses after maternal age adjustments. The relationships between each of the four microRNA genotypes and each of the six clinical parameters of the RPL patients (plasma homocysteine and folate levels, natural killer cell number, platelet count, prothrombin time, and, activated partial thromboplastin time) were analyzed using multiple linear regression analyses.

**Results:**

Our results suggest that weak associations between decreased RPL risk and the genotypes of *miR-27a* (AG and AG+GG), combination genotype of *miR-27a*/*miR-423* (AG/GC), and haplotypes of *miR-27a*/*miR-423*/*miR-449b*/*miR-605* (G-C-A-G) and *miR-27a*/*miR-449b*/*miR-605* (G-A-G), whereas weak associations between increased RPL risk and genotypes of *miR-449b* (GG and AG+GG), combination genotypes of *miR-423*/*miR-449b* (CC/GG and CA/AG), *miR-449b*/*miR-605* (AG/AG), haplotypes of *miR-27a*/*miR-423*/*miR-449b*/*miR-605* (A-C-G-A, A-A-A-G, and G-C-G-G), *miR-27a*/*miR-423*/*miR-449b* (A-C-G), *miR-27a*/*miR-449b*/*miR-605* (A-A-G, A-G-A, and G-G-G), *miR-423*/*miR-449b*/*miR-605* (C-G-G and A-A-G), and *miR-423*/*miR-449b* (C-G and A-A). The genotypes of *miR-27a* (AG and AG+GG) also showed significant contributions to the prediction of folate levels in RPL patients.

**Conclusions:**

The study showed associations between miRNA polymorphisms (*miR-27a* rs895819 and *miR-449b* rs10061133) and RPL development, and between the miRNA polymorphism (*miR-27a* rs895819) and plasma folate levels.

## Introduction

Recurrent pregnancy loss (RPL) or recurrent spontaneous abortion has been defined as the occurrence of at least two consecutive pregnancy losses prior to the 20th week of gestation [1, 2]. RPL occurs in approximately 1% of all pregnancies; however, the etiology for more than half of the RPLs remains undetermined [3]. Genetic variation has been suggested one of the contributing factors leading to RPL and a number of single nucleotide polymorphisms (SNPs) have been reported to be associated with RPL [4]. MicroRNAs (miRNAs) are short (approximately 22 nt) noncoding RNA molecules regulating expression of target genes at the post-transcriptional level by translational repression or messenger RNA degradation [5]. Several studies recently reported the associations between miRNA polymorphisms and RPL [6–9]. One study identified two SNPs in miR-125a altering the production of miR-125a which was subsequently associated with an elevated risk for RPL in the Han Chinese women [8]. Another study reported an association between two pre-miRNA polymorphisms (miR-196a2 and miR-499) and the occurrence of RPL in Korean females [9], which was supported in Iranian women [6]. The most recent study identified a polymorphism in the coding region of *miR-423* contributing to an increase in the expression of mature *miR-423* associated with RPL in the Han Chinese population [7]. Several miRNAs that are considered important during pregnancy were chosen for this study because of their elevated expression (*miR-27a*), decreased expression in the endometrium and in trophoblasts (*miR-423*), lower expression during endometriosis (*miR-449b*), and involvement in pregnancy loss via the p53 network (*miR-605*) [10–13]. In this study, we determined the susceptibility to RPL associated with genetic variants of miRNAs associated with placental or fetal development.

## Materials and methods

### Study participants

The study group consisted of 387 females, 33.21 ± 4.55 years of age [mean age ± standard deviation (SD)] and a body mass index (BMI) of 21.49 ± 3.84 (± SD) who were diagnosed as idiopathic RPL patients with at least two consecutive pregnancy losses prior to the 20th week of gestation according to the definitions of infertility and recurrent pregnancy loss by American Society for Reproductive Medicine [1]. These patients were enrolled in a study at the Infertility Medical Center of CHA Bundang Medical Center from March 1999 to February 2012. Among the RPL patients, none had a history of smoking or alcohol use. RPL patients due to anatomical, hormonal, chromosomal (patients or their spouses), infectious, autoimmune, or thrombotic causes are excluded from the study. The age-matched control group consisted of 225 females, 33.43 ± 5.89 years of age (mean age ± SD) and with a BMI of 21.68 ± 3.451 (± SD), each of whom had regular menstrual cycles, had a history of naturally conceived pregnancy at least once, had no history of pregnancy loss or karyotype 46,XX, and who were recruited from the CHA Bundang Medical Center. All patients and controls were Korean. The institutional review board of CHA Bundang Medical Center approved the study, and all patients provided written informed consent.

### Genotyping

Genomic DNA was extracted from non-coagulated peripheral blood using the G-DEX blood extraction kit (Intron, Seongnam, Korea). The nucleotide changes were determined by the polymerase chain reaction (PCR)-restriction fragment length polymorphism analyses using the isolated genomic DNA as a template. Primer sequences for PCR amplification of each polymorphism were as follows: *miR-27a*A>G [rs895819], forward 5'-GAA CTT AGC CAC TGT GAA CAC CAC TTG G-3' and reverse 5'-TTG CTT CCT GTC ACA AAT CAC ATT G-3' (the mismatch sequence is underlined) [14]; *miR-423*C>A [rs6505162], forward 5'-GTA CAT TTT CCC GGA TGG AA-3' and reverse 5'-GGG AGA AAC TCA AGC GCC G-3'; *miR-449b*A>G [rs10061113], forward 5'-GGT ATC CAG AGC ACT TCA TTG ACA-3' and reverse 5'-ACC TGA ATC AGG TAG GCA GTG TCT-3'; and *miR-605*A>G [rs2043556], forward 5'-AGA GCA GTT ACG CCA CAT GA-3' and reverse 5'-GCC TTC TCC TTG GGA AAA AC-3'. We performed a restriction enzyme digestion at 37°C for 16 hours using *Dra*III (New England BioLabs, Ipswich, MA, USA) for the *miR-27a* polymorphism, *Bsr*FI for the *miR-423* polymorphism, *Bsm*AI for the *miR-449b* polymorphism, and *Hin*fI for the *miR-605* polymorphism. We confirmed the genotyping of the four sites by sequencing 10% of the samples.

### Assessment of homocysteine, folate, total cholesterol, and urate concentrations, and blood coagulation

Blood samples from RPL patients were collected during pregnancy. Plasma homocysteine, folate, total cholesterol, and urate concentrations, and blood coagulation factors were measured in RPL patients after fasting for 12 hours. Homocysteine levels (6.98 ± 2.10 μM) were measured using a fluorescence polarization immunoassay and the Abbott IMx analyzer (Abbott Laboratories, Abbott Park, IL, USA). Folate levels (14.21 ± 11.94 ng/mL) were determined using a competitive immunoassay with ACS:180 (Bayer Diagnostics, Tarrytown, NY, USA). Total cholesterol (187.73 ± 49.42 mg/dL) and urate levels (3.80 ± 0.84 mg/dL) were determined using commercially available enzymatic colorimetric tests (Roche Diagnostics, Mannheim, Germany). Platelet (PLT) counts, prothrombin time (PT), and activated partial thromboplastin time (aPTT) were measured to assess blood coagulation. PLT counts (255.43 ± 59.22 10^3^ cells/μL) were measured using a Sysmex XE2100 automated hematology analyzer (Sysmex, Kobe, Japan). PT (11.58 ± 0.85 seconds) and aPTT (32.24 ± 4.33 seconds) were measured using an automated photo-optical coagulometer (ACL TOP; Mitsubishi Chemical Medience, Tokyo, Japan).

### Preparation of blood samples and estimation of peripheral natural killer (NK) cells

Peripheral blood mononuclear cells (PBMCs) were isolated from whole blood using a cell preparation tube containing sodium citrate (Becton-Dickinson, Franklin Lakes, NJ, USA). To obtain monocytes, viable PBMCs were frozen in 80% fetal bovine serum (FBS; Lonza, Cologne, Germany), 10% dimethyl sulfoxide (Sigma-Aldrich, St. Louis, MO, USA), and 10% RPMI 1640 media (Life Technologies, Carlsbad, CA, USA) in liquid nitrogen. After thawing, the PBMCs were cultured in RPMI 1640 media supplemented with 10% FBS, 50 mg/mL gentamicin sulfate (Lonza), 50 μM 2-mercaptoethanol (Sigma-Aldrich), and 2 mM glutamine (Life Technologies). The cells were washed twice with phosphate buffered saline (Welgene, Seoul, Korea) and then resuspended in RPMI 1640 media containing 10% FBS, 1% Minimal Essential Media with nonessential amino acids (Life Technologies), and 1% sodium pyruvate (Life Technologies) at a density of 1 × 10^6^ cells/mL, and incubated overnight as described above. All NK cell assays were performed after 16–20 hours of incubation.

To determine the absolute number of NK cells, 200 mL of diluted blood was incubated for 20 minutes on ice with phycoerythrin-conjugated anti-CD56 and peridinin chlorophyll protein-conjugated anti-CD3 monoclonal antibodies (BD Biosciences, San Jose, CA, USA). Then, 20,000 fluorescein isothiocyanate conjugated beads were added, and the blood sample was subjected to erythrocyte lysis using FACS Lysing Solution (BD Biosciences). The samples were analyzed on a flow cytometer using the BD FACSCalibur (BD Biosciences). The NK cell number of the diluted blood samples was calculated as (NK cells/mL sample) = [(CD56^+^/CD3^-^ cell count)/(bead count)] × 100. This FACS-based NK cell count required 0.1 mL of whole blood per tested condition.

### Statistical analysis

The differences in four microRNAs (*miR-27a*A>G, *miR-423*C>A, *miR-499b*A>G, and *miR-605*A>G) genotype and haplotype frequencies between patients and normal controls were compared using Fisher’s exact test and logistic regression analyses. Allele frequencies were estimated to identify deviations from the Hardy-Weinberg equilibrium (HWE). Adjusted odds ratios (AOR) and 95% confidence interval (CI) were estimated as a measure of the strength of association between genotypes and RPL risk. Multiple comparison tests were adjusted by using the false discovery rate (FDR) correction, and associations with an FDR-adjusted *P* value < .05 were considered statistically significant [15].

Gene-gene interactions among SNP loci were analyzed with multifactor dimensionality reduction (MDR) using MDR software, version 2.0 (www.epistasis.org) [16–18]. Based on the MDR identification of the most significant models using the best maximized cross validation value, the best multilocus combinations were determined. HAPSTAT software, version 3.0 (www.bios.unc.edu/Èlin/hapstat/) was used to estimate haplotype frequencies for polymorphisms that were determined by MDR analyses to have strong synergistic effects. Statistical analyses were performed using GraphPad Prism software, version 4.0 (GraphPad, San Diego, CA, USA) and StatsDirect software, version 2.4.4 (StatsDirect, Altrincham, UK). The statistical significance was set at *P* <0.05. The relationship between each of the four microRNA genotypes and each of the six clinical variables of the RPL patients (plasma homocysteine, folate, NK cell, PLT, PT, and aPTT which may contribute to an even balance of coagulation and fibrinolysis during pregnancy) was analyzed using multiple linear regression analyses. Regression models were examined for the six clinical variables with grades (10 levels) as dependent variables and the microRNA polymorphisms as independent variables. The differences in plasma homocysteine, folate, PLT, PT, aPTT, and NK cells as a function of the four microRNA genotypes and combination genotypes were evaluated by one-way analysis of variance and independent two-sample *t*-tests.

## Results

The demographic characteristics and clinical profiles of RPL patients and control subjects are shown in Table 1. The two groups were matched for age and BMI. Platelet numbers were significantly higher in the patient group than in the control group. The genotype and allele frequencies of the four miRNA SNPs in females with RPL and controls are shown in Table 2. All genotypes in the study cases were in HWE. In Table 2, the *miR-27a* A>G polymorphism was significantly associated with a risk of RPL (AA vs. AG: AOR = 0.654; 95% CI = 0.456–0.937; AA vs. AG+GG: AOR = 0.682; 95% CI = 0.484–0.960); however, there was no association after adjustment for multiple tests using the FDR correction. When RPL patients were stratified according to the occurrence of consecutive recurrent pregnancy losses (RPL = 2 and ≥ 3 vs. all RPL patients with RPL ≥ 2), there was an association between the *miR -27a* A>G polymorphism and RPL risk in the RPL ≥ 3 subgroup alone (AA vs. AG: AOR = 0.611; 95% CI = 0.404–0.923; AA vs. AG+GG: AOR = 0.639; 95% CI = 0.432–0.945). However, the associations were not statistically significant after adjustment for multiple tests using the FDR correction. The *miR-449b* A>G polymorphism was significantly associated with RPL risk (AA vs. GG: AOR = 2.069; 95% CI = 1.033–.4.146; AA vs AG+GG: AOR = 1.406; 95% CI = 1.011–1.955). However, the association was not significant after adjustment for multiple tests using the FDR correction. Using combination analyses (Table 3), the AG/CC (AOR = 0.579; 95% CI = 0.366–0.917) combined genotype for *miR-27a*/*miR-423* was associated with a lower RPL risk compared with reference genotypes when variant genotypes were located in the *miR-27a* loci. In addition, CC/GG (AOR = 2.888; 95% CI = 1.116–7.470), CA/AG (AOR = 1.925; 95% CI = 1.110–3.338) for the *miR-423*/*miR-449b*, and AG/AG (AOR = 1.804; 95% CI = 1.067–3.052) for the *miR-449b*/*miR-605* were associated with an increased RPL risk compared with reference genotypes when variant genotypes were located in the loci of *miR-423*, *miR-449b*, and *miR-605*. These results were consistent with associations between a RPL risk and individual microRNA genotypes; however, each association was not significant after the FDR correction for multiple comparisons, suggesting a weak association. Haplotype-based analyses of the four microRNA polymorphisms for gene-gene interactions are shown in S1 Table (all possible allele combinations) and Table 4 (allele combinations suggesting associations with RPL). Interaction models suggested by the MDR were evaluated using haplotype-based analyses. Among the models of the four polymorphic loci, the G-C-A-G haplotype (OR = 0.525; 95% CI = 0.321–0.859) was associated with a decreased RPL risk whereas three haplotypes, A-C-G-A (OR = 1.870; 95% CI = 1.178–2.968), A-A-A-G (OR = 2.429; 95% = 1.153–5.114), and G-C-G-G (OR = 3.214; 95% CI = 1.441–7.172), were associated with an increased RPL risk. Among the models of the three polymorphic loci, one haplotype, G-A-G of *miR-27a/miR-449b/miR-605* (OR = 0.625; 95% CI = 0.402–0.972), was associated with a reduced RPL risk, whereas haplotypes A-C-G of *miR-27a/miR-423/miR-449b* (OR = 1.498; 95% CI = 1.030–2.179); A-A-G, A-G-A, and G-G-G of *miR-27a/miR-449b/miR-605* (OR = 1.526; 95% CI = 1.037–2.244; OR = 1.649; 95% CI = 1.086–2.504; OR = 3.089; 95% CI = 1.401–6.809), C-G-G, and A-A-G of *miR-423/miR-449b/miR-605* (OR = 1.773; 95% CI = 1.110–2.833; OR = 2.253; 95% CI = 1.203–4.220) were associated with an increased RPL risk. Among the models of the two polymorphic loci, two haplotypes, C-G and A-A of *miR-423/miR-449b* (OR = 1.518; 95% CI = 1.134–2.031), were associated with a higher RPL risk. The G-G-G of *miR-27a/miR-449b/miR-605* and C-G and A-A of *miR-423/miR-449b* haplotypes remained significant after adjustment for multiple tests using the FDR correction. Multiple linear regression analyses of clinical variables in Korean RPL patients according to the quintiles of clinical variables are shown in Table 5. The AG and AG+GG genotypes of the *miR-27a* polymorphism showed significant contributions to the prediction of folate levels in RPL patients, with regression coefficients of 1.069 and 0.788, respectively. Although the differences in plasma homocysteine, folate, PLT, PT, aPTT, and NK cell number in relation to the four microRNA genotypes and haplotypes were evaluated by one-way analysis of variance and independent two sample *t*-tests, no significant difference was found (S2 and S3 Tables).

**Table 1:** Clinical profiles of RPL patients and control subjects.

Characteristics [normal range]	Control subjects (n = 225)	RPL patients (n = 387)	*P*^e^
Age (years)	33.43 ± 5.78	33.21 ± 4.55	0.963
BMI (kg/m^2^)	21.68 ± 3.45	21.49 ± 3.84	0.668
Previous pregnancy losses	NA	3.02 ± 1.51	
Live birth	1.82 ± 0.75	NA	
Average gestational weeks	39.30 ± 1.63	7.36 ± 1.93	<0.001
RPL<14 weeks	NA	98.8%	
CD56 NK cells (%) [5.6~31]	NA	18.26 ± 7.99	
Homocysteine (μM) [< 12]	NA	6.98 ± 2.10	
Folate (ng/mL) [3.45–13.77]	NA	14.21 ± 11.94	
Total cholesterol (mg/dL) [< 200]	NA	187.73 ± 49.42	
Urate (mg/dL) [2.4–6.1]	NA	3.80 ± 0.84	
Platelet (10^3^/μL) [130–370]	239.11 ± 64.58^a^	255.43 ± 59.22^c^	0.010
aPTT (seconds) [28.2–39.2]	33.39 ± 3.82^b^	32.24 ± 4.33^d^	0.058
Prothrombin time (seconds) [10.6–12.8]	NA	11.58 ± 0.85	

Note: RPL = recurrent pregnancy loss; BMI = body mass index; NK = natural killer; NA = not applicable; aPTT = activated partial thromboplastin time; values are mean ± standard deviation unless otherwise noted.

^a^ Platelet count of 178 control subjects;

^b^ Activated partial thromboplastin time of 63 control subjects;

^c^ Platelet count of 205 RPL patients;

^d^ Activated partial thromboplastin time of 210 RPL patients;

^e^ *P* values were calculated using the two-sided *t*-test for continuous variables.

**Table 2:** Genotype frequencies of *miR-27a*A>G, *miR-423*C>A, *miR-499b*A>G, and *miR-605*A>G polymorphisms in Korean RPL patients and control subjects.

Genotypes	Controls (n = 225)	RPL ≥ 2 (n = 387)	AOR (95% CI)	*P*^a^	*P*^b^	RPL = 2 (n = 181)	AOR (95% CI)	*P*^a^	*P*^b^	RPL≥3 (n = 206)	AOR (95% CI)	*P*^a^	*P*^b^
***miR -27a* rs895819 A>G**													
AA	74 (32.9)	166 (42.9)	1.000 (reference)			75 (41.4)	1.000 (reference)			91 (44.2)	1.000 (reference)		
AG	120 (53.3)	170 (43.9)	0.654 (0.456–0.937)	0.021	0.084	81 (44.8)	0.703 (0.459–1.079)	0.107	0.214	89 (43.2)	0.611 (0.404–0.923)	0.019	0.076
GG	31 (13.8)	51 (13.2)	0.774 (0.457–1.311)	0.341	0.469	25 (13.8)	0.843 (0.453–1.570)	0.591	0.843	26 (12.6)	0.724 (0.395–1.330)	0.298	0.397
Dominant (AA vs. AG + GG)			0.682 (0.484–0.960)	0.028	0.086		0.732 (0.488–1.099)	0.133	0.177		0.639 (0.432–0.945)	0.025	0.100
Recessive (AA + AG vs. GG)			0.980 (0.603–1.591)	0.933	0.933		1.027 (0.579–1.824)	0.926	0.926		0.948 (0.539–1.667)	0.853	0.853
HWE *P*	0.110	0.474											
***miR-423* rs6505162 C>A**													
CC	149 (66.2)	232 (59.9)	1.000 (reference)			104 (57.5)	1.000 (reference)			128 (62.1)	1.000 (reference)		
CA	65 (28.9)	130 (33.6)	1.277 (0.889–1.836)	0.186	0.248	67 (37.0)	1.447 (0.946–2.214)	0.088	0.176	63 (30.6)	1.143 (0.750–1.741)	0.535	0.535
AA	11 (4.9)	25 (6.5)	1.421 (0.678–2.980)	0.352	0.469	10 (5.5)	1.245 (0.508–3.051)	0.632	0.843	15 (7.3)	1.594 (0.704–3.608)	0.264	0.352
Dominant (CC vs. CA + AA)			1.303 (0.924–1.838)	0.131	0.175		1.423 (0.948–2.134)	0.088	0.153		1.213 (0.816–1.804)	0.340	0.453
Recessive (CC + CA vs. AA)			1.332 (0.642–2.763)	0.442	0.589		1.109 (0.459–2.678)	0.818	0.975		1.562 (0.698–3.492)	0.278	0.433
HWE *P*	0.268	0.246											
***miR-449b* rs10061133 A>G**													
AA	119 (52.9)	172 (44.4)	1.000 (reference)			82 (45.3)	1.000 (reference)			90 (43.7)	1.000 (reference)		
AG	94 (41.8)	179 (46.3)	1.318 (0.936–1.856)	0.113	0.226	82 (45.3)	1.276 (0.847–1.922)	0.245	0.291	97 (47.1)	1.361 (0.918–2.020)	0.125	0.250
GG	12 (5.3)	36 (9.3)	2.069 (1.033–4.146)	0.040	0.160	17 (9.4)	2.116 (0.956–4.686)	0.065	0.260	19 (9.2)	2.047 (0.943–4.444)	0.070	0.280
Dominant (AA vs. AG + GG)			1.406 (1.011–1.955)	0.043	0.086		1.373 (0.926–2.037)	0.115	0.198		1.443 (0.986–2.110)	0.059	0.118
Recessive (AA + AG vs. GG)			1.837 (0.935–3.611)	0.078	0.312		1.920 (0.889–4.146)	0.097	0.388		1.788 (0.845–3.784)	0.129	0.516
HWE *P*	0.231	0.283											
***miR-605* rs2043556 A>G**													
AA	107 (47.6)	164 (42.4)	1.000 (reference)			76 (42.0)	1.000 (reference)			88 (42.7)	1.000 (reference)		
AG	91 (40.4)	183 (47.3)	1.360 (0.785–2.355)	0.273	0.273	87 (48.1)	1.433 (0.735–2.794)	0.291	0.291	96 (46.6)	1.282 (0.681–2.415)	0.441	0.589
GG	27 (12.0)	40 (10.3)	1.035 (0.600–1.785)	0.903	0.903	18 (9.9)	1.064 (0.547–2.069)	0.855	0.898	22 (10.7)	1.008 (0.537–1.892)	0.981	0.981
Dominant (AA vs. AG + GG)			1.183 (0.704–1.987)	0.525	0.525		1.232 (0.654–2.319)	0.519	0.519		1.135 (0.624–2.064)	0.678	0.678
Recessive (AA + AG vs. GG)			0.809 (0.582–1.126)	0.210	0.420		0.795 (0.535–1.181)	0.256	0.512		0.826 (0.564–1.209)	0.325	0.650
HWE *P*	0.267	0.289											

Note: RPL = recurrent pregnancy loss; AOR = adjusted odds ratio by the age of participants; OR = odds ratio; CI = confidence interval; FDR = false discovery rate; HWE = Hardy-Weinberg equilibrium.

^***a***^ Fisher’s exact test;

^***b***^ FDR-adjusted *P* value

**Table 3:** Combination analyses of *miR-27a*A>G, *miR-423*C>A, *miR-499b*A>G, and *miR-605*A>G polymorphisms in Korean RPL patients and control subjects.

Genotypes	Controls (n = 225)	RPL (n = 387)	AOR (95% CI)	*P*^a^	*P*^b^
***miR-27a/miR-423***					
AA/CC	44 (19.6)	95 (24.5)	1.000 (reference)		
AA/CA	27 (12.0)	59 (15.3)	1.013 (0.568–1.808)	0.964	0.979
AA/AA	5 (2.2)	12 (3.1)	1.015 (0.332–3.101)	0.979	0.979
AG/CC	86 (38.2)	106 (27.4)	0.579 (0.366–0.917)	0.020	0.160
AG/CA	29 (12.9)	55 (14.2)	0.881 (0.496–1.566)	0.666	0.979
AG/AA	4 (1.8)	9 (2.3)	1.030 (0.300–3.533)	0.963	0.979
GG/CC	19 (8.4)	31 (8.0)	0.771 (0.391–1.519)	0.452	0.979
GG/CA	9 (4.0)	16 (4.1)	0.789 (0.321–1.942)	0.607	0.979
GG/AA	2 (0.9)	4 (1.0)	0.853 (0.149–4.893)	0.859	0.979
***miR-27a/miR-449b***					
AA/AA	38 (16.9)	76 (19.6)	1.000 (reference)		
AA/AG	35 (15.6)	73 (18.7)	1.042 (0.595–1.825)	0.886	0.886
AA/GG	3 (1.3)	17 (4.4)	2.739 (0.753–9.967)	0.126	0.336
AG/AA	63 (28.0)	78 (20.2)	0.608 (0.364–1.017)	0.058	0.332
AG/AG	50 (22.2)	78 (20.2)	0.774 (0.456–1.312)	0.341	0.546
AG/GG	6 (2.6)	14 (3.6)	1.107 (0.389–3.150)	0.848	0.886
GG/AA	18 (8.0)	18 (4.7)	0.506 (0.234–1.092)	0.083	0.332
GG/AG	9 (4.0)	28 (7.2)	1.548 (0.663–3.614)	0.312	0.546
GG/GG	3 (1.3)	5 (1.3)	0.840 (0.190–3.717)	0.819	0.886
***miR-27a/miR-605***					
AA/AA	38 (16.9)	75 (19.4)	1.000 (reference)		
AA/AG	30 (13.3)	70 (18.1)	1.173 (0.657–2.095)	0.590	0.703
AA/GG	8 (3.6)	21 (5.4)	1.336 (0.541–3.300)	0.530	0.703
AG/AA	55 (24.4)	68 (17.6)	0.625 (0.368–1.060)	0.081	0.324
AG/AG	49 (21.8)	84 (21.7)	0.902 (0.530–1.534)	0.703	0.703
AG/GG	15 (6.7)	18 (4.7)	0.616 (0.279–1.360)	0.230	0.613
GG/AA	14 (6.2)	21 (5.4)	0.767 (0.348–1.689)	0.510	0.703
GG/AG	12 (5.3)	29 (7.5)	1.204 (0.552–2.627)	0.641	0.703
GG/GG	4 (1.8)	1 (0.3)	0.113 (0.012–1.075)	0.058	0.324
***miR-423/miR-449b***					
CC/AA	79 (35.1)	101 (26.1)	1.000 (reference)		
CC/AG	64 (28.4)	109 (28.2)	1.320 (0.861–2.024)	0.203	0.325
CC/GG	6 (2.7)	22 (5.7)	2.888 (1.116–7.470)	0.029	0.116
CA/AA	35 (15.6)	55 (14.2)	1.249 (0.741–2.104)	0.404	0.539
CA/AG	25 (11.1)	62 (16.0)	1.925 (1.110–3.338)	0.020	0.116
CA/GG	5 (2.2)	13 (3.4)	2.065 (0.705–6.047)	0.186	0.325
AA/AA	5 (2.2)	16 (4.1)	2.462 (0.862–7.030)	0.092	0.245
AA/AG	5 (2.2)	8 (2.1)	1.219 (0.383–3.885)	0.738	0.838
AA/GG	1 (0.4)	1 (0.3)	0.748 (0.046–12.193)	0.838	0.838
***miR-423/miR-605***					
CC/AA	71 (31.6)	98 (25.3)	1.000 (reference)		
CC/AG	59 (26.2)	111 (28.7)	1.386 (0.892–2.155)	0.147	0.520
CC/GG	19 (8.4)	23 (5.9)	0.874 (0.443–1.726)	0.698	0.768
CA/AA	30 (13.3)	55 (14.2)	1.328 (0.774–2.278)	0.303	0.587
CA/AG	28 (12.4)	60 (15.5)	1.557 (0.903–2.684)	0.111	0.520
CA/GG	7 (3.1)	15 (3.9)	1.548 (0.599–4.000)	0.367	0.587
AA/AA	6 (2.7)	11 (2.8)	1.307 (0.461–3.712)	0.615	0.768
AA/AG	4 (1.8)	12 (3.1)	2.173 (0.673–7.016)	0.195	0.520
AA/GG	1 (0.4)	2 (0.5)	1.441 (0.127–16.305)	0.768	0.768
***miR-449b/miR-605***					
AA/AA	57 (25.3)	74 (19.1)	1.000 (reference)		
AA/AG	49 (21.8)	83 (21.4)	1.305 (0.796–2.139)	0.291	0.582
AA/GG	13 (5.8)	15 (3.9)	0.906 (0.398–2.064)	0.815	0.815
AG/AA	46 (20.4)	75 (19.4)	1.255 (0.758–2.078)	0.377	0.604
AG/AG	35 (15.6)	82 (21.2)	1.804 (1.067–3.052)	0.028	0.222
AG/GG	13 (5.8)	22 (5.7)	1.295 (0.599–2.800)	0.512	0.604
GG/AA	4 (1.8)	15 (3.9)	2.939 (0.923–9.361)	0.068	0.273
GG/AG	7 (3.1)	18 (4.7)	1.882 (0.731–4.847)	0.190	0.507
GG/GG	1 (0.4)	3 (0.8)	2.094 (0.210–20.890)	0.529	0.604

Note: RPL = recurrent pregnancy loss; AOR = adjusted odds ratio; CI = confidence interval.

^a^ Fisher’s exact test;

^b^ FDR-adjusted *P* value.

**Table 4:** Haplotype-based analyses of *miR-27a*A>G, *miR-423*C>A, *miR-499b*A>G, and *miR-605*A>G polymorphisms in Korean RPL patients and control subjects.

Haplotypes	Controls (2n = 450), n (%)	RPL (2n = 774), n (%)	OR (95% CI)	*P*^a^	*P*^b^
***miR-27a/miR-423/miR-449b/miR-605***					
A-C-A-A	115 (25.7)	161 (20.7)	1.000 (reference)		
A-C-G-A	34 (7.5)	89 (11.5)	1.870 (1.178–2.968)	0.008	0.052
A-A-A-G	10 (2.1)	34 (4.5)	2.429 (1.153–5.114)	0.020	0.073
G-C-A-G	49 (10.9)	36 (4.6)	0.525 (0.321–0.859)	0.010	0.052
G-C-G-G	8 (1.8)	36 (4.6)	3.214 (1.441–7.172)	0.004	0.052
***miR-27a/miR-423/miR-449b***					
A-C-A	158 (35.2)	252 (32.5)	1.000 (reference)		
A-C-G	54 (12.1)	129 (16.7)	1.498 (1.030–2.179)	0.035	0.242
***miR-27a/miR-449b/miR-605***					
A-A-A	145 (32.3)	223 (28.8)	1.000 (reference)		
A-A-G	52 (11.5)	122 (15.8)	1.526 (1.037–2.244)	0.032	0.064
A-G-A	41 (9.1)	104 (13.5)	1.649 (1.086–2.504)	0.019	0.064
G-A-G	52 (11.6)	50 (6.5)	0.625 (0.402–0.972)	0.037	0.064
G-G-G	8 (1.7)	38 (4.9)	3.089 (1.401–6.809)	0.005	0.036
***miR-423/miR-449b/miR-605***					
C-A-A	181 (40.1)	264 (34.1)	1.000 (reference)		
C-G-G	29 (6.4)	75 (9.7)	1.773 (1.110–2.833)	0.017	0.058
A-A-G	14 (3.2)	46 (5.9)	2.253 (1.203–4.220)	0.011	0.058
***miR-423/miR-449b***					
C-A	271 (60.2)	392 (50.7)	1.000 (reference)		
C-G	92 (20.5)	202 (26.1)	1.518 (1.134–2.031)	0.005	0.015
A-A	61 (13.6)	131 (16.9)	1.485 (1.056–2.088)	0.023	0.035

Note: RPL = recurrent pregnancy loss; OR = odds ratio; CI = confidence interval; ORs and 95% CIs of each haplotype combination were calculated with reference to frequencies of all others using Fisher’s exact test.

^a^ Fisher’s exact test;

^b^ FDR-adjusted *P* value.

**Table 5:** Multiple linear regression analyses of clinical variables in Korean RPL patients according to the quintiles of clinical variables.

Genotypes	Hcy decile^a^ (μM)			Folate decile^b^ (ng/mL)			NK cells decile^c^ (%)			PLT decile^d^ (10^3^/μL)			PT decile^e^ (seconds)			aPTT decile^f^ (seconds)		
	n = 282	Coef	*P*	n = 223	Coef	*P*	n = 134	Coef	*P*	n = 205	Coef	*P*	n = 208	Coef	*P*	n = 210	Coef	*P*
***miR -27a* rs895819 A>G**																		
AA	121 (42.9)	(ref.)		95 (42.6)	(ref)		59 (44.0)	(ref.)		90 (43.9)	(ref.)		93 (44.7)	(ref.)		94 (44.8)	(ref.)	
AG	126 (44.7)	-0.532	0.151	102 (45.7)	1.069	0.008	61 (45.5)	0.190	0.724	90 (43.9)	0.045	0.918	91 (43.8)	-0.215	0.624	92 (43.8)	-0.364	0.395
GG	35 (12.4)	-0.074	0.895	26 (11.7)	-0.301	0.640	14 (10.4)	-0.030	0.973	25 (12.2)	0.296	0.650	24 (11.5)	1.111	0.102	24 (11.4)	0.176	0.793
Dominant (AA vs. AG+GG)		-0.426	0.219		0.788	0.045		0.162	0.753		0.098	0.809		0.050	0.904		-0.271	0.500
Recessive (AA + AG vs. GG)		0.214	0.681		-0.842	0.164		-0.020	0.981		0.269	0.665		1.242	0.053		0.308	0.625
***miR-423* rs6505162 C>A**																		
CC	167 (59.2)	(ref.)		135 (60.5)	(ref.)		81 (60.4)	(ref.)		126 (61.5)	(ref.)		129 (62.0)	(ref.)		130 (61.9)	(ref.)	
CA	96 (34.0)	0.320	0.392	73 (32.7)	-0.287	0.503	42 (31.3)	-0.560	0.322	64 (31.2)	-0.636	0.150	64 (30.8)	-0.515	0.255	65 (31.0)	-0.199	0.644
AA	19 (6.7)	0.449	0.501	15 (6.7)	-0.558	0.476	11 (8.2)	-0.417	0.665	15 (7.3)	-1.323	0.082	15 (7.2)	0.926	0.238	15 (7.1)	1.099	0.157
Dominant (CC vs. CA + AA)		0.340	0.331		-0.334	0.402		-0.527	0.312		-0.763	0.065		-0.237	0.574		0.046	0.911
Recessive (CC + CA vs. AA)		0.298	0.663		-0.449	0.561		-0.228	0.807		-1.087	0.160		1.106	0.162		1.175	0.128
***miR-449b* rs10061133 A>G**																		
AA	143 (50.7)	(ref.)		133 (59.6)	(ref.)		80 (59.7)	(ref.)		109 (53.2)	(ref.)		116 (55.8)	(ref.)		117 (55.7)	(ref.)	
AG	119 (42.2)	0.356	0.316	79 (35.4)	-0.002	0.997	49 (36.6)	-0.203	0.710	77 (37.6)	0.300	0.485	74 (35.6)	0.077	0.859	75 (35.7)	0.148	0.726
GG	20 (7.1)	-0.352	0.611	11 (4.9)	0.359	0.698	5 (3.7)	-0.842	0.550	19 (9.3)	1.169	0.105	18 (8.7)	-0.443	0.564	18 (8.6)	-1.052	0.163
Dominant (AA vs. AG + GG)		0.260	0.449		0.048	0.904		-0.251	0.632		0.453	0.265		-0.046	0.912		-0.083	0.836
Recessive (AA + AG vs. GG)		-0.520	0.438		0.386	0.666		-0.762	0.573		1.011	0.146		-0.472	0.518		-1.083	0.129
***miR-605* rs2043556 A>G**																		
AA	126 (44.7)	(ref.)		96 (43.0)	(ref.)		58 (43.3)	(ref.)		87 (42.4)	(ref.)		89 (42.8)	(ref.)		90 (42.9)	(ref.)	
AG	130 (46.1)	-0.148	0.806	108 (48.4)	-0.840	0.264	67 (50.0)	1.741	0.105	96 (46.8)	-0.485	0.478	100 (48.1)	0.394	0.589	101 (48.1)	-0.177	0.805
GG	26 (9.2)	0.231	0.717	19 (8.5)	-1.012	0.145	9 (6.7)	1.654	0.112	22 (10.7)	-0.415	0.564	19 (9.1)	1.043	0.174	19 (9.0)	0.620	0.403
Dominant (AA vs. AG + GG)		0.040	0.946		-0.945	0.172		1.707	0.093		-0.452	0.488		0.698	0.326		0.209	0.764
Recessive (AA + AG vs. GG)		0.352	0.307		-0.409	0.299		0.223	0.666		-0.026	0.949		0.693	0.094		0.759	0.059

Note: RPL = recurrent pregnancy loss; Hcy = homocysteine; NK cells = natural killer cells; PLT = platelet count; PT = prothrombin time; aPTT = activated partial thromboplastin time; R^2^ = coefficient of determination; Coef = regression coefficients; Ref = reference.

^a^ Homocysteine 10 quintiles: Hcy≤4.69, 4.69<Hcy≤5.46, 5.46<Hcy≤5.9, 5.9<Hcy≤6.31, 6.31<Hcy≤6.73, 6.73<Hcy≤7.2, 7.2<Hcy≤7.55, 7.55<Hcy≤8.2, 8.2<Hcy≤9.22, Hcy>9.22;

^b^ Folate 10 quintiles: folate≤5.34, 5.34<folate≤6.70, 6.70<folate≤7.79, 7.79<folate≤9.62, 9.62<folate≤11.16, 11.16<folate≤13.25, 13.25<folate≤16.59, 16.59<folate≤19.65, 19.65<folate≤22.54, folate>22.54;

^c^ NK cells 10 quintiles: NK cells≤9, 9<NK cells≤11, 11<NK cells≤13, 13<NK cells≤15, 15<NK cells≤17, 17<NK cells≤19.5, 19.5<NK cells≤21.5, 21.5<NK cells≤25, 25<NK cells≤30, NK cells>30;

^d^ Platelet count 10 quintiles: PLT≤187, 187<PLT≤207, 207<PLT≤224, 224<PLT≤237, 237<PLT≤251, 251<PLT≤266, 266<PLT≤279, 279<PLT≤302, 302<PLT≤329, PLT>329; prothrombin time 10 quintiles:

^e^ PT≤10.5, 10.5<PT≤11, 11<PT≤11.2, 11.2<PT≤11.4, 11.4<PT≤11.6, 11.6<PT≤11.75, 11.75<PT≤11.9, 11.9<PT≤12.25, 12.25<PT≤12.6, PT>12.6;

^f^ Activated partial thromboplastin time: aPTT≤26.8, 26.8<aPTT≤28.4, 28.4<aPTT≤29.6, 29.6<aPTT≤30.75, 30.75<aPTT≤31.9, 31.9<aPTT≤33.15, 33.15<aPTT≤34.4, 34.4<aPTT≤36, 36<aPTT≤37.5, aPTT>37.5

## Discussion

The effects of miRNA polymorphisms on pregnancy loss have been reported in a limited number of studies [6–9]. These studies investigated the roles of *miR-125*, *miR-196a2*, *miR-499* and *miR-423* polymorphisms that were supported by functional evidence showing disruption of mature microRNA production and its downstream target gene [6–9, 19]. Because more evidence in support of the functional importance of miRNAs in pregnancy has been reported, we determined whether the four miRNA SNPs associated with placental or fetal development played a role in pregnancy loss [14, 20–27]. Our results showed an association between the *miR-27a* variant G allele and a lower RPL risk, and an association between the *miR-449b* variant G allele and a higher RPL risk. The effects of variant alleles of *miR-27a* and *miR-449b* were also suggested using combination and haplotype-based analyses. Multiple linear regression analyses of clinical variables in Korean RPL patients revealed statistically significant relationships between the *miR-27a* genotypes and plasma folate levels. Functional analyses indicated that the variant genotypes of *miR-27a*, AG, and GG might be responsible for the elevated *miR-27a* levels [14], and it has been speculated that the elevated *miR-27a* levels contribute to the increased folate concentration that is protective against RPL [20]. However, the possible effects of *mir-27a* on RPL mediated by folate require further investigation to confirm this hypothesis. The *miR-449b* was chosen for the study because it is one of the four miRNAs that was downregulated in hatched blastocysts [21]. However, the effect of rs10061133 A>G of *miR-449b* has rarely been reported, and there have been contradictory reports on its effects, depending on the cell type and gene expression pattern [24]. In our study, we found that the GG and AG+GG genotypes of *miR-449b* were associated with an increased risk of RPL. We, therefore, hypothesize that the GG and AG+GG genotypes of *miR-449b* rs10061133 affect the risk of RPL risk by modulating the expression of mature *miR-449b* [24, 27].

The *miR-423* rs6505162 polymorphism was chosen for the present study because it was reported to be differentially regulated in placental injury [25], and rs6505162 in pre-*miR-423* affects the mature miRNA expression by increasing miRNA expression with the variant A allele [26]. However, in our study, we did not find a statistically significant association between *miR-423* genotypes and RPL risk. Notably, a recent study in the Han Chinese population also failed to identify an association between the *miR-423* genotypes and RPL risk; however, the study found an association between *miR-423* alleles and RPL, with functional evidence showing that the minor A allele contributed to an increased expression of mature *miR-423* [7]. An explanation for the different results may be related to the different allele frequencies. The *miR-605* rs2043556 A>G polymorphism was included in the study because the variant G allele of *miR-605* caused a 2.6-fold reduction in the processing levels of *miR-605* [23], and *miR-605* was significantly dysregulated in placentas after exposure to Bisphenol A, which may disrupt endocrine levels [22]. However, we did not find a significant association between the *miR-605* polymorphism and a risk of RPL. A miR-125a polymorphism involving one nucleotide mutation in the pri-miR-125a coding region related to RPL was not included in the study because the location and nomenclature of the polymorphism were not clear, although the A>G mutation reduced the expression of mature miR-125a [8].

There were some limitations in our study. First, this case-control study identified an association between SNPs in miRNAs in RPL development, but it did not establish a cause-and-effect relationship. What we identified, therefore, warrants functional studies to elucidate the pathogenesis related to RPL. Second, we reported a significant relationship between *miR-27a* genotypes and plasma folate levels in Korean RPL patients, and we speculated that elevated *miR-27a* levels contributed to the increased folate levels. However, further functional studies are necessary to directly investigate the effect of *mir-27a* on RPL when mediated by folate levels. Third, we did not determine the effect of miRNA polymorphisms on miRNA expression in the placenta, which might have contributed to the potential roles of miRNA during the peri-implantation and early pregnancy periods.

Accumulation of findings on post-transcriptional regulation of gene expression by miRNAs and their involvements in trophoblast differentiation, proliferation, and angiogenesis during the developments of placenta or fetus have broaden our perspectives on the roles of miRNA and microRNA machinery gene polymorphisms during the pathogenesis of idiopathic RPL since three recent reports on the association of miRNA polymorphisms with RPL in Han-Chinese and Korean women [6–9, 12, 19, 28–32]. In the present study, we identified associations between miRNA polymorphisms (*miR-27a*A>G and *miR-449b*A>G) and RPL risk in Korean females, and identified a relationship between miRNA polymorphism (*miR-27a*A>G) and plasma folate levels. However, what we identified in our study warrants further investigation to elucidate the underlying mechanism in which miRNA polymorphisms modulate folate levels and RPL development.

## Supporting information

S1 Table Haplotype-based analyses of *miR-27aA>G*, *miR-423C>A*, *miR-499bA>G*, and *miR-605A>G* polymorphisms in Korean RPL patients and control subjects for all possible allele combinations (combinations of four sites, three sites, two sites are listed respectively). (DOCX)

S2 Table Differences of clinical parameters according to the four microRNA polymorphisms in RPL patients. (DOCX)

S3 Table Differences in clinical parameters according to the haplotypes of the four microRNA polymorphisms in RPL patients. (DOCX)

## Abbreviations

AOR: adjusted odds ratio
CI: confidence interval
FDR: false discovery rate
HWE: hardy-weinberg equilibrium
MDR: multifactor dimensionality reduction
PCR: polymerase chain reaction
RFLP: restriction fragment length polymorphism
RPL: recurrent pregnancy loss
SNP: single nucleotide polymorphism
