# Supplementary material for: miR-27a and miR-449b polymorphisms associated with a risk of idiopathic recurrent pregnancy loss
Source: PLoS One. 2017 May 10;12(5):e0177160. doi: 10.1371/journal.pone.0177160 (PMC5425187; doi:10.1371/journal.pone.0177160)
Supplement: S1 Table — (DOCX) [file pone.0177160.s001.docx]

**S1 Table**

**Haplotype-based analyses of *miR-27a*A>G, *miR-423*C>A, *miR-499b*A>G, and *miR-605*A>G polymorphisms in Korean RPL patients and control subjects for all possible allele combinations (combinations of four sites, three sites, two sites are listed respectively).**

|  | | | | | | | |  |
| --- | --- | --- | --- | --- | --- | --- | --- | --- |
| **Characteristics of four sites** | **Overall** | **Control (n= 225)** | **Case (n=387)** | **Control  (2n=450)** | **Case  (2n=774)** | **OR (95% CI)** | ***P*^a^** | ***P*^b^** |
| ***miR-27a/miR-423/miR-449b/miR-605*** | | | |  |  |  |  |  |
| A-C-A-A | 0.232 | 0.257 | 0.207 | 115 (25.7) | 161 (20.7) | 1.000 (reference) |  |  |
| A-C-A-G | 0.102 | 0.095 | 0.118 | 43 (9.5) | 91 (11.8) | 1.512 (0.979 - 2.334) | 0.062 | 0.187 |
| A-C-G-A | 0.097 | 0.075 | 0.115 | 34 (7.5) | 89 (11.5) | 1.870 (1.178 - 2.968) | 0.008 | 0.052 |
| A-C-G-G | 0.053 | 0.043 | 0.051 | 19 (4.3) | 39 (5.1) | 1.393 (0.772 - 2.512) | 0.271 | 0.369 |
| A-A-A-A | 0.071 | 0.067 | 0.077 | 30 (6.7) | 60 (7.7) | 1.429 (0.867 - 2.354) | 0.162 | 0.346 |
| A-A-A-G | 0.038 | 0.021 | 0.045 | 10 (2.1) | 34 (4.5) | 2.429 (1.153 - 5.114) | 0.020 | 0.073 |
| A-A-G-A | 0.022 | 0.016 | 0.022 | 7 (1.6) | 17 (2.2) | 1.735 (0.697 - 4.319) | 0.237 | 0.355 |
| A-A-G-G | 0.018 | 0.029 | 0.015 | 13 (2.9) | 12 (1.5) | 0.659 (0.290 - 1.498) | 0.320 | 0.400 |
| G-C-A-A | 0.134 | 0.142 | 0.139 | 64 (14.2) | 107 (13.9) | 1.194 (0.807 - 1.766) | 0.374 | 0.432 |
| G-C-A-G | 0.077 | 0.109 | 0.046 | 49 (10.9) | 36 (4.6) | 0.525 (0.321 - 0.859) | 0.010 | 0.052 |
| G-C-G-A | 0.057 | 0.069 | 0.045 | 31 (6.9) | 35 (4.5) | 0.807 (0.470 - 1.383) | 0.434 | 0.465 |
| G-C-G-G | 0.032 | 0.018 | 0.047 | 8 (1.8) | 36 (4.7) | 3.214 (1.441 - 7.172) | 0.004 | 0.052 |
| G-A-A-A | 0.035 | 0.040 | 0.031 | 18 (4.0) | 24 (3.1) | 0.952 (0.494 - 1.836) | 0.884 | 0.884 |
| G-A-A-G | 0.011 | 0.007 | 0.013 | 3 (0.7) | 10 (1.3) | 2.381 (0.641 - 8.844) | 0.195 | 0.355 |
| G-A-G-A | 0.020 | 0.013 | 0.025 | 6 (1.3) | 19 (2.5) | 2.262 (0.876 - 5.840) | 0.092 | 0.229 |
| G-A-G-G | 0.004 | 0.000 | 0.005 | 0 (0.0) | 4 (0.5) | 6.437 (0.343 -120.727) | 0.213 | 0.355 |
| Note: RPL = recurrent pregnancy loss; OR = odds ratio; CI = confidence interval; ORs and 95% CIs of each haplotype combination were calculated with reference to frequencies of all others using Fisher’s exact test.  ^a^Fisher’s exact test;  ^b^FDR-adjusted *P* value | | | | | | | |  |

|  | | | | | | | | |  |  |  |
| --- | --- | --- | --- | --- | --- | --- | --- | --- | --- | --- | --- |
| **Characteristics of three sites** | **Overall** | **Control (n= 225)** | **Case (n=387)** | **Control  (2n=450)** | **Case  (2n=774)** | **OR (95% CI)** | ***P*^a^** | ***P*^b^** |  |  |  |
| ***miR-27a/miR-423/miR-449b*** | | | |  |  |  |  |  |  |  |  |
| A-C-A | 0.334 | 0.352 | 0.325 | 158 (35.2) | 252 (32.5) | 1.000 (reference) |  |  |  |  |  |
| A-C-G | 0.151 | 0.121 | 0.167 | 54 (12.1) | 129 (16.7) | 1.498 (1.030 - 2.179) | 0.035 | 0.242 |  |  |  |
| A-A-A | 0.108 | 0.088 | 0.121 | 40 (8.8) | 94 (12.1) | 1.473 (0.968 - 2.243) | 0.079 | 0.276 |  |  |  |
| A-A-G | 0.038 | 0.041 | 0.036 | 18 (4.1) | 28 (3.6) | 0.975 (0.522 - 1.822) | 1.000 | 1.000 |  |  |  |
| G-C-A | 0.209 | 0.250 | 0.183 | 112 (25.0) | 142 (18.3) | 0.795 (0.579 - 1.092) | 0.167 | 0.292 |  |  |  |
| G-C-G | 0.088 | 0.084 | 0.093 | 38 (8.4) | 72 (9.3) | 1.188 (0.765 - 1.846) | 0.506 | 0.708 |  |  |  |
| G-A-A | 0.047 | 0.048 | 0.047 | 21 (4.8) | 36 (4.7) | 1.075 (0.606 - 1.908) | 0.885 | 1.000 |  |  |  |
| G-A-G | 0.025 | 0.017 | 0.029 | 7 (1.7) | 22 (2.9) | 1.971 (0.823 - 4.721) | 0.164 | 0.292 |  |  |  |
| ***miR-27a/miR-423/miR-605*** | | | |  |  |  |  |  |  |  |  |
| A-C-A | 0.328 | 0.333 | 0.325 | 150 (33.3) | 251 (32.5) | 1.000 (reference) |  |  |  |  |  |
| A-C-G | 0.155 | 0.137 | 0.165 | 62 (13.7) | 128 (16.5) | 1.234 (0.857 - 1.777) | 0.272 | 0.583 |  |  |  |
| A-A-A | 0.093 | 0.083 | 0.099 | 37 (8.3) | 77 (9.9) | 1.244 (0.800 - 1.934) | 0.378 | 0.583 |  |  |  |
| A-A-G | 0.056 | 0.049 | 0.060 | 22 (4.9) | 46 (6.0) | 1.250 (0.723 - 2.159) | 0.497 | 0.583 |  |  |  |
| G-C-A | 0.191 | 0.208 | 0.181 | 94 (20.8) | 140 (18.1) | 0.890 (0.640 - 1.239) | 0.500 | 0.583 |  |  |  |
| G-C-G | 0.109 | 0.128 | 0.097 | 58 (12.8) | 75 (9.7) | 0.773 (0.519 - 1.151) | 0.219 | 0.583 |  |  |  |
| G-A-A | 0.055 | 0.054 | 0.056 | 24 (5.4) | 43 (5.6) | 1.071 (0.625 - 1.835) | 0.892 | 0.892 |  |  |  |
| G-A-G | 0.014 | 0.008 | 0.018 | 4 (0.8) | 14 (1.8) | 2.092 (0.676 - 6.473) | 0.221 | 0.583 |  |  |  |
| ***miR-27a/miR-449b/miR-605*** | | | |  |  |  |  |  |  |  |  |
| A-A-A | 0.304 | 0.323 | 0.288 | 145 (32.3) | 223 (28.8) | 1.000 (reference) |  |  |  |  |  |
| A-A-G | 0.139 | 0.115 | 0.158 | 52 (11.5) | 122 (15.8) | 1.526 (1.037 - 2.244) | 0.032 | 0.064 |  |  |  |
| A-G-A | 0.118 | 0.091 | 0.135 | 41 (9.1) | 104 (13.5) | 1.649 (1.086 - 2.504) | 0.019 | 0.064 |  |  |  |
| A-G-G | 0.071 | 0.074 | 0.068 | 33 (7.4) | 53 (6.8) | 1.044 (0.645 - 1.692) | 0.903 | 1.000 |  |  |  |
| G-A-A | 0.167 | 0.184 | 0.165 | 83 (18.4) | 128 (16.5) | 1.003 (0.709 - 1.418) | 1.000 | 1.000 |  |  |  |
| G-A-G | 0.089 | 0.116 | 0.065 | 52 (11.6) | 50 (6.5) | 0.625 (0.402 - 0.972) | 0.037 | 0.064 |  |  |  |
| G-G-A | 0.078 | 0.080 | 0.073 | 36 (8.0) | 56 (7.3) | 1.011 (0.633 - 1.615) | 1.000 | 1.000 |  |  |  |
| G-G-G | 0.035 | 0.017 | 0.049 | 8 (1.7) | 38 (4.9) | 3.089 (1.401 - 6.809) | 0.005 | 0.036 |  |  |  |
| ***miR-423/miR-449b/miR-605*** | | | |  |  |  |  |  |  |  |  |
| C-A-A | 0.366 | 0.401 | 0.341 | 181 (40.1) | 264 (34.1) | 1.000 (reference) |  |  |  |  |  |
| C-A-G | 0.177 | 0.201 | 0.165 | 91 (20.1) | 128 (16.5) | 0.964 (0.694 - 1.340) | 0.867 | 0.867 |  |  |  |
| C-G-A | 0.154 | 0.141 | 0.165 | 63 (14.1) | 127 (16.5) | 1.382 (0.968 - 1.974) | 0.076 | 0.177 |  |  |  |
| C-G-G | 0.085 | 0.064 | 0.097 | 29 (6.4) | 75 (9.7) | 1.773 (1.110 - 2.833) | 0.017 | 0.058 |  |  |  |
| A-A-A | 0.106 | 0.104 | 0.110 | 47 (10.4) | 85 (11.0) | 1.240 (0.828 - 1.856) | 0.312 | 0.437 |  |  |  |
| A-A-G | 0.050 | 0.032 | 0.059 | 14 (3.2) | 46 (5.9) | 2.253 (1.203 - 4.220) | 0.011 | 0.058 |  |  |  |
| A-G-A | 0.041 | 0.032 | 0.044 | 14 (3.2) | 34 (4.4) | 1.665 (0.868 - 3.192) | 0.161 | 0.282 |  |  |  |
| A-G-G | 0.022 | 0.026 | 0.019 | 12 (2.6) | 15 (1.9) | 0.857 (0.392 - 1.974) | 0.693 | 0.809 |  |  |  |
| Note: RPL = recurrent pregnancy loss; OR = odds ratio; CI = confidence interval; ORs and 95% CIs of each haplotype combination were calculated with reference to frequencies of all others using Fisher’s exact test.  ^a^Fisher’s exact test;  ^b^FDR-adjusted *P* value | | | | | | | |  |  |  |  |

|  | | | | | | | | |  |  |  |
| --- | --- | --- | --- | --- | --- | --- | --- | --- | --- | --- | --- |
| **Characteristics of two sites** | **Overall** | **Control (n= 225)** | **Case (n=387)** | **Control  (2n=450)** | **Case  (2n=774)** | **OR (95% CI)** | ***P*^a^** | ***P*^b^** |  |  |  |
| ***miR-27a/miR-423*** | | | |  |  |  |  |  |  |  |  |
| A-C | 0.485 | 0.474 | 0.491 | 213 (47.4) | 380 (49.1) | 1.000 (reference) |  |  |  |  |  |
| A-A | 0.147 | 0.129 | 0.157 | 58 (12.9) | 122 (15.7) | 1.179 (0.827 - 1.681) | 0.374 | 0.561 |  |  |  |
| G-C | 0.297 | 0.333 | 0.276 | 150 (33.3) | 214 (27.6) | 0.800 (0.612 - 1.045) | 0.115 | 0.345 |  |  |  |
| G-A | 0.071 | 0.065 | 0.075 | 29 (6.5) | 58 (7.5) | 1.121 (0.696 - 1.805) | 0.719 | 0.719 |  |  |  |
| ***miR-27a/miR-449b*** | | | |  |  |  |  |  |  |  |  |
| A-A | 0.443 | 0.4391 | 0.4455 | 198 (43.9) | 345 (44.6) | 1.000 (reference) |  |  |  |  |  |
| A-G | 0.189 | 0.1632 | 0.2031 | 73 (16.3) | 157 (20.3) | 1.234 (0.889 - 1.714) | 0.217 | 0.325 |  |  |  |
| G-A | 0.256 | 0.2987 | 0.2302 | 134 (29.9) | 178 (23.0) | 0.762 (0.574 - 1.013) | 0.068 | 0.204 |  |  |  |
| G-G | 0.113 | 0.0991 | 0.1212 | 45 (9.9) | 94 (12.1) | 1.199 (0.807 - 1.781) | 0.427 | 0.427 |  |  |  |
| ***miR-27a/miR-605*** | | | |  |  |  |  |  |  |  |  |
| A-A | 0.421 | 0.4166 | 0.4243 | 187 (41.7) | 328 (42.4) | 1.000 (reference) |  |  |  |  |  |
| A-G | 0.210 | 0.1857 | 0.2243 | 84 (18.6) | 174 (22.4) | 1.181 (0.861 - 1.621) | 0.338 | 0.411 |  |  |  |
| G-A | 0.245 | 0.2612 | 0.2359 | 118 (26.1) | 183 (23.6) | 0.884 (0.660 - 1.185) | 0.411 | 0.411 |  |  |  |
| G-G | 0.123 | 0.1366 | 0.1155 | 61 (13.7) | 89 (11.6) | 0.832 (0.573 - 1.207) | 0.339 | 0.411 |  |  |  |
| ***miR-423/miR-449b*** | | | |  |  |  |  |  |  |  |  |
| C-A | 0.543 | 0.602 | 0.507 | 271 (60.2) | 392 (50.7) | 1.000 (reference) |  |  |  |  |  |
| C-G | 0.239 | 0.205 | 0.261 | 92 (20.5) | 202 (26.1) | 1.518 (1.135 - 2.031) | 0.005 | 0.015 |  |  |  |
| A-A | 0.156 | 0.136 | 0.169 | 61 (13.6) | 131 (16.9) | 1.485 (1.056 - 2.088) | 0.023 | 0.035 |  |  |  |
| A-G | 0.063 | 0.058 | 0.064 | 26 (5.8) | 49 (6.4) | 1.303 (0.790 - 2.149) | 0.322 | 0.322 |  |  |  |
| ***miR-423/ miR-605*** | | | |  |  |  |  |  |  |  |  |
| C-A | 0.520 | 0.543 | 0.506 | 244 (54.3) | 392 (50.6) | 1.000 (reference) |  |  |  |  |  |
| C-G | 0.262 | 0.264 | 0.261 | 119 (26.4) | 202 (26.1) | 1.057 (0.801 - 1.394) | 0.725 | 0.725 |  |  |  |
| A-A | 0.147 | 0.135 | 0.154 | 61 (13.5) | 119 (15.4) | 1.214 (0.858 - 1.719) | 0.296 | 0.444 |  |  |  |
| A-G | 0.071 | 0.058 | 0.079 | 26 (5.8) | 61 (7.9) | 1.460 (0.898 - 2.375) | 0.156 | 0.444 |  |  |  |
| ***miR-449b/miR-605*** | | | |  |  | 0 (0.0) |  |  |  |  |  |
| A-A | 0.472 | 0.506 | 0.451 | 228 (50.6) | 349 (45.1) | 1.000 (reference) |  |  |  |  |  |
| A-G | 0.227 | 0.232 | 0.225 | 104 (23.2) | 174 (22.5) | 1.093 (0.814 - 1.468) | 0.600 | 0.600 |  |  |  |
| G-A | 0.195 | 0.172 | 0.209 | 77 (17.2) | 162 (20.9) | 1.374 (1.000 - 1.890) | 0.056 | 0.164 |  |  |  |
| G-G | 0.106 | 0.091 | 0.115 | 41 (9.1) | 89 (11.5) | 1.418 (0.945 - 2.128) | 0.109 | 0.164 |  |  |  |
| Note: RPL = recurrent pregnancy loss; OR = odds ratio; CI = confidence interval; ORs and 95% CIs of each haplotype combination were calculated with reference to frequencies of all others using Fisher’s exact test.  ^a^Fisher’s exact test;  ^b^FDR-adjusted *P* value | | | | | | | |  |  |  |  |
