# Supplementary material for: miR-27a and miR-449b polymorphisms associated with a risk of idiopathic recurrent pregnancy loss
Source: PLoS One. 2017 May 10;12(5):e0177160. doi: 10.1371/journal.pone.0177160 (PMC5425187; doi:10.1371/journal.pone.0177160)
Supplement: S2 Table — (DOCX) [file pone.0177160.s002.docx]

**S2 Table**

**Differences of clinical parameters according to the four microRNA polymorphisms in RPL patients.**

| **Genotypes** | **Hcy (μM)** | **FA (ng/mL)** | **PLT (10^3^/µl)** | **aPTT** | **NK cell (%)** | **PT (seconds)** |
| --- | --- | --- | --- | --- | --- | --- |
|  | **Mean ± SD** | **Mean ± SD** | **Mean ± SD** | **Mean ± SD** | **Mean ± SD** | **Mean ± SD** |
| ***miR-27a* rs895819 A>G** |  |  |  |  |  |  |
| AA | 7.11 ± 2.09 | 13.91 ± 15.15 | 245.66 ± 63.21 | 32.59 ± 4.43 | 18.06 ± 8.16 | 11.55 ± 0.85 |
| AG | 6.87 ± 2.22 | 15.23 ± 9.25 | 250.74 ± 62.20 | 32.22 ± 4.01 | 18.37 ± 7.97 | 11.56 ± 0.88 |
| GG | 6.87 ± 1.63 | 11.30 ± 6.91 | 243.21 ± 59.96 | 33.34 ± 4.41 | 18.57 ± 7.89 | 11.81 ± 0.79 |
| **P^a^** | 0.635 | 0.311 | 0.654 | 0.394 | 0.965 | 0.375 |
| ***miR-423* rs6505162 C>A** |  |  |  |  |  |  |
| CC | 6.87 ± 1.91 | 14.88 ± 13.41 | 250.53 ± 64.19 | 32.38 ± 3.91 | 18.86 ± 8.38 | 11.62 ± 0.81 |
| CA | 7.10 ± 2.35 | 13.65 ± 9.96 | 245.90 ± 60.43 | 32.42 ± 4.67 | 17.42 ± 7.30 | 11.46 ± 0.94 |
| AA | 7.28 ± 2.33 | 10.94 ± 4.10 | 227.96 ± 45.01 | 33.92 ± 5.02 | 16.97 ± 7.82 | 11.78 ± 0.90 |
| ***P^a^*** | 0.556 | 0.428 | 0.245 | 0.472^b^ | 0.550 | 0.309 |
| ***miR-449b* rs10061133 A>G** |  |  |  |  |  |  |
| AA | 6.79 ± 1.69 | 13.50 ± 8.15 | 244.63 ± 54.66 | 32.58 ± 4.18 | 18.60 ± 8.67 | 11.60 ± 0.88 |
| AG | 7.18 ± 2.29 | 15.43 ± 16.90 | 248.57 ± 68.48 | 32.58 ± 4.00 | 17.99 ± 7.10 | 11.60 ± 0.80 |
| GG | 7.08 ± 3.25 | 14.16 ± 7.80 | 265.72 ± 74.68 | 31.60 ± 5.54 | 15.40 ± 4.56 | 11.36 ± 0.98 |
| ***P^a^*** | 0.311 | 0.532 | 0.220 | 0.580 | 0.661 | 0.525 |
| ***miR-605* rs2043556 G>A** |  |  |  |  |  |  |
| AA | 7.14 ± 2.28 | 12.94 ± 8.51 | 248.43 ± 61.90 | 32.94 ± 4.29 | 18.49 ± 7.51 | 11.71 ± 0.91 |
| AG | 6.86 ± 2.00 | 14.27 ± 10.64 | 242.75 ± 57.86 | 32.15 ± 4.35 | 18.59 ± 8.38 | 11.51 ± 0.79 |
| GG | 6.75 ± 1.55 | 20.10 ± 25.44 | 264.64 ± 76.30 | 32.17 ± 3.28 | 14.22 ± 7.74 | 11.35 ± 0.95 |
| ***P^a^*** | 0.466 | 0.384^b^ | 0.115 | 0.311 | 0.294 | 0.131 |

Note: RPL = recurrent pregnancy loss; Hcy = homocysteine; FA = folate; PLT = platelet count; aPTT = activated partial thromboplastin time; NK = natural killer; PT = prothrombin time.
^a^Calculated using ANOVA;
^b^Calculated using the Kruskal-Wallis test.
