# Supplementary material for: miR-27a and miR-449b polymorphisms associated with a risk of idiopathic recurrent pregnancy loss
Source: PLoS One. 2017 May 10;12(5):e0177160. doi: 10.1371/journal.pone.0177160 (PMC5425187; doi:10.1371/journal.pone.0177160)
Supplement: S3 Table — (DOCX) [file pone.0177160.s003.docx]

**S3 Table**

**Differences in clinical parameters according to the haplotypes of the four microRNA polymorphisms in RPL patients.**

| **Haplotypes** | **Hcy (μM)** | **FA (ng/mL)** | **PLT (10^3^/µl)** | **aPTT** | **NK cell (%)** | **PT (seconds)** |
| --- | --- | --- | --- | --- | --- | --- |
|  | **Mean ± SD** | **Mean ± SD** | **Mean ± SD** | **Mean ± SD** | **Mean ± SD** | **Mean ± SD** |
| ***miR-27/miR-423*** |  |  |  |  |  |  |
| A/CC | 6.94 ± 1.74 | 15.14 ± 18.59 | 246.60 ± 66.29 | 32.85 ± 4.34 | 18.09 ± 8.48 | 11.64 ± 0.81 |
| AA/CA | 7.16 ± 2.32 | 12.74 ± 9.45 | 246.41 ± 60.64 | 31.69 ± 4.52 | 18.66 ± 7.78 | 11.35 ± 0.80 |
| AA/AA | 8.19 ± 3.08 | 10.11 ± 3.38 | 236.69 ± 55.27 | 33.98 ± 4.51 | 15.95 ± 8.47 | 11.70 ± 1.29 |
| AG/CC | 6.81 ± 2.15 | 15.26 ± 8.73 | 254.21 ± 62.60 | 31.86 ± 3.55 | 18.86 ± 8.52 | 11.53 ± 0.79 |
| AG/CA | 7.06 ± 2.54 | 15.71 ± 11.10 | 247.00 ± 64.70 | 32.53 ± 4.47 | 16.91 ± 7.25 | 11.51 ± 1.14 |
| AG/AA | 6.55 ± 0.54 | 12.81 ± 3.77 | 219.50 ± 17.77 | 34.61 ± 5.78 | 20.75 ± 6.02 | 11.94 ± 0.39 |
| GG/CC | 6.86 ± 1.52 | 12.55 ± 7.22 | 246.40 ± 65.90 | 32.96 ± 3.70 | 21.78 ± 7.53 | 11.89 ± 0.82 |
| [GG/CA](file:///C:\Downloads\고기한\MIR%20RSA%20통계%20.xls#RANGE!A2#RANGE!A2) | 7.04 ± 1.89 | 9.90 ± 6.25 | 240.07 ± 44.51 | 34.69 ± 5.55 | 14.00 ± 4.76 | 11.74 ± 0.79 |
| GG/AA | 6.26 ± 1.70 | 3.75 ± NA | 182.00 ± NA | 27.75 ± NA | 8.00 ± NA | 11.30 ± NA |
| ***P^a^*** | 0.798 | 0.766 | 0.778 | 0.188 | 0.630 | 0.587 |
| ***miR-27/miR-449b*** |  |  |  |  |  |  |
| AA/AA | 7.10 ± 1.76 | 12.76 ± 8.04 | 238.19 ± 54.97 | 32.46 ± 4.25 | 18.05 ± 9.08 | 11.52 ± 0.82 |
| AA/AG | 7.35 ± 2.50 | 16.56 ± 24.94 | 248.21 ± 74.02 | 33.13 ± 3.82 | 18.64 ± 6.89 | 11.73 ± 0.81 |
| AA/GG | 6.08 ± 1.45 | 12.89 ± 7.64 | 272.67 ± 50.08 | 31.55 ± 6.69 | 14.33 ± 3.51 | 11.16 ± 1.03 |
| AG/AA | 6.47 ± 1.65 | 15.30 ± 8.28 | 246.88 ± 54.92 | 32.31 ± 3.97 | 18.86 ± 8.28 | 11.59 ± 0.96 |
| AG/AG | 7.22 ± 2.27 | 15.06 ± 10.96 | 254.53 ± 63.60 | 32.02 ± 4.20 | 17.68 ± 7.77 | 11.48 ± 0.74 |
| AG/GG | 8.19 ± 4.92 | 15.67 ± 8.60 | 261.32 ± 108.07 | 32.50 ± 3.75 | 17.00 ± 7.07 | 11.64 ± 0.98 |
| GG/AA | 6.97 ± 1.29 | 8.57 ± 5.52 | 254.83 ± 52.46 | 34.19 ± 4.71 | 20.00 ± 9.59 | 12.00 ± 0.64 |
| GG/AG | 6.64 ± 1.68 | 14.03 ± 7.27 | 227.55 ± 68.48 | 32.84 ± 3.89 | 17.14 ± 6.18 | 11.59 ± 0.95 |
| GG/GG | 7.88 ± 2.86 | NA | 251.75 ± 52.12 | 28.73 ± 1.38 | NA | 11.80 ± 0.71 |
| ***P^a^*** | 0.411^b^ | 0.581 | 0.435 | 0.565 | 0.979 | 0.503 |
| ***miR-27/miR-605*** |  |  |  |  |  |  |
| AA/AA | 7.25 ± 2.31 | 10.37 ± 4.83 | 247.69 ± 57.56 | 32.43 ± 4.58 | 18.10 ± 6.45 | 11.62 ± 0.85 |
| AA/AG | 7.02 ± 1.94 | 14.90 ± 13.79 | 233.78 ± 57.78 | 32.92 ± 4.61 | 18.56 ± 9.49 | 11.52 ± 0.78 |
| AA/GG | 6.93 ± 1.76 | 22.58 ± 32.98 | 281.60 ± 93.87 | 31.98 ± 3.16 | 15.86 ± 7.99 | 11.34 ± 1.12 |
| AG/AA | 7.09 ± 2.42 | 15.58 ± 10.67 | 253.16 ± 65.09 | 32.88 ± 3.74 | 18.29 ± 7.85 | 11.72 ± 1.04 |
| AG/AG | 6.76 ± 2.17 | 14.67 ± 7.92 | 245.15 ± 56.76 | 31.57 ± 4.22 | 19.12 ± 8.02 | 11.47 ± 0.77 |
| AG/GG | 6.41 ± 1.27 | 16.71 ± 9.33 | 262.96 ± 71.23 | 32.48 ± 3.96 | 8.50 ± 3.54 | 11.36 ± 0.73 |
| GG/AA | 6.98 ± 1.76 | 11.47 ± 6.00 | 232.30 ± 64.61 | 35.07 ± 4.95 | 21.00 ± 10.33 | 12.03 ± 0.58 |
| GG/AG | 6.75 ± 1.59 | 11.17 ± 7.72 | 257.09 ± 61.16 | 32.03 ± 3.85 | 16.75 ± 5.52 | 11.63 ± 0.91 |
| GG/GG | 7.47 ± NA | NA | 228.67 ± 25.37 | 31.83 ± 1.07 | NA | NA |
| ***P^a^*** | 0.950 | 0.107^b^ | 0.160 | 0.302 | 0.668 | 0.496 |
| ***miR -423/miR-449b*** |  |  |  |  |  |  |
| CC/AA | 6.66 ± 1.59 | 14.64 ± 8.23 | 251.20 ± 55.98 | 32.22 ± 3.52 | 19.30 ± 8.89 | 11.63 ± 0.78 |
| CC/AG | 6.90 ± 1.79 | 15.26 ± 19.37 | 246.78 ± 70.13 | 32.67 ± 3.88 | 18.73 ± 8.03 | 11.65 ± 0.77 |
| CC/GG | 8.10 ± 3.70 | 14.89 ± 8.83 | 268.84 ± 85.50 | 32.10 ± 6.58 | 15.40 ± 4.56 | 11.37 ± 1.14 |
| CA/AA | 7.01 ± 1.90 | 12.30 ± 8.51 | 233.03 ± 51.14 | 33.01 ± 4.99 | 18.26 ± 8.30 | 11.54 ± 1.02 |
| CA/AG | 7.48 ± 2.80 | 16.61 ± 12.67 | 255.49 ± 69.26 | 31.70 ± 4.66 | 16.19 ± 5.51 | 11.35 ± 0.85 |
| CA/GG | 5.56 ± 1.81 | 12.19 ± 4.84 | 275.92 ± 57.07 | 31.44 ± 4.63 | NA | 11.36 ± 0.87 |
| AA/AA | 6.77 ± 1.35 | 10.61 ± 3.52 | 232.0 ± 49.16 | 33.84 ± 5.48 | 14.96 ± 8.57 | 11.64 ± 1.06 |
| AA/AG | 8.33 ± 3.32 | 11.61 ± 5.48 | 234.0 ± 39.21 | 35.65 ± 4.04 | 20.50 ± 5.57 | 12.14 ± 0.58 |
| AA/GG | 5.56 ± NA | NA | 179.50 ± 3.54 | 29.23 ± 2.09 | NA | 11.30 ± NA |
| ***P^a^*** | 0.394 | 0.8 | 0.137 | 0.389 | 0.657 | 0.709 |
| ***miR -423/miR-605*** |  |  |  |  |  |  |
| CC/AA | 7.07 ± 2.29 | 13.19 ± 7.61 | 252.43 ± 66.25 | 32.61± 3.83 | 18.48 ± 8.16 | 11.66 ± 0.82 |
| CC/AG | 6.70 ± 1.57 | 15.0 ± 11.90 | 241.54 ± 58.19 | 32.08 ± 4.20 | 19.37 ± 8.67 | 11.63 ± 0.77 |
| CC/GG | 6.71 ± 1.39 | 22.37 ± 31.21 | 272.87 ± 70.87 | 32.67 ± 2.84 | 17.50 ± 9.47 | 11.36 ± 0.95 |
| CA/AA | 7.24 ± 2.10 | 12.65 ± 10.78 | 245.08 ± 54.71 | 32.92 ± 4.76 | 18.01 ± 6.54 | 11.65 ± 1.11 |
| CA/AG | 7.05 ± 2.63 | 13.99 ± 8.91 | 247.02 ± 59.17 | 32.06 ± 4.66 | 18.19 ± 7.58 | 11.29 ± 0.83 |
| CA/GG | 6.64 ± 1.92 | 17.14 ± 13.69 | 244.18 ± 91.60 | 31.88 ± 4.63 | 8.33 ± 2.52 | 11.58 ± 0.69 |
| AA/AA | 7.27 ± 3.09 | 12.17 ± 4.32 | 219.70 ± 32.68 | 35.87 ± 5.38 | 20.50 ± 5.57 | 12.34 ± 0.58 |
| [AA/AG](file:///C:\Documents%20and%20Settings\Owner\Local%20Settings\Temporary%20Internet%20Files\Content.MSO\2AE51B5.xls#RANGE!A2) | 7.25 ± 1.16 | 8.53 ± 3.35 | 231.90 ± 50.09 | 33.31 ± 4.54 | 14.34 ± 9.92 | 11.51 ± 0.33 |
| AA/GG | 7.48 ± 2.1 | 13.92 ± 1.85 | 249.50 ± 92.63 | 29.7 ± 2.51 | 16.5 ± 6.36 | 10.65 ± 1.91 |
| ***P^a^*** | 0.931 | 0.718 | 0.301 | 0.337 | 0.536 | 0.1 |
| ***miR-449b/miR-605*** |  |  |  |  |  |  |
| AA/AA | 6.95 ± 1.74 | 12.98 ± 7.47 | 249.35 ± 54.13 | 33.20 ± 4.29 | 18.40 ± 7.50 | 11.80 ± 0.89 |
| AA/AG | 6.65 ± 1.65 | 14.05 ± 9.04 | 237.49 ± 56.58 | 32.43 ± 4.21 | 19.24 ± 9.64 | 11.51 ± 0.78 |
| AA/GG | 6.77 ± 1.67 | 12.82 ± 6.09 | 254.70 ± 46.20 | 30.80 ± 3.12 | 15.71 ± 8.08 | 11.27 ± 1.17 |
| AG/AA | 7.24 ± 2.37 | 12.96 ± 10.04 | 248.12 ± 68.40 | 33.26 ± 4.10 | 19.12 ± 8.02 | 11.71 ± 0.83 |
| AG/AG | 7.18 ± 2.40 | 14.42 ± 13.63 | 238.87 ± 53.60 | 31.48 ± 4.00 | 17.72 ± 5.98 | 11.53 ± 0.82 |
| AG/GG | 6.90 ± 1.39 | 35.90 ± 42.46 | 276.00 ± 95.56 | 34.16 ± 2.47 | 9.00 ± 4.24 | 11.46 ± 0.59 |
| GG/AA | 7.91 ± 4.52 | 12.43 ± 8.94 | 244.23 ± 78.88 | 30.60 ± 4.57 | 15.75 ± 5.19 | 11.30 ± 1.16 |
| GG/AG | 6.71 ± 2.12 | 16.23 ± 6.51 | 293.29 ± 63.87 | 33.04 ± 6.74 | 14.00 ± NA | 11.44 ± 0.76 |
| GG/GG | 4.61 ± NA | NA | 202.00 ± NA | NA | NA | NA |
| ***P^a^*** | 0.626 | 0.570 | 0.138^b^ | 0.078 | 0.661 | 0.424 |

Note: RPL = recurrent pregnancy loss; Hcy = homocysteine; FA = folate; PLT = platelet count; aPTT = activated partial thromboplastin time; NK = natural killer; PT = prothrombin time.
^a^Calculated using ANOVA;

^b^Calculated using the Kruskal-Wallis test.
